# Changes in Household Wealth Over the Process of Widowhood Across European Countries

**DOI:** 10.1093/geronb/gbae116

**Published:** 2024-07-16

**Authors:** Nicole Kapelle, Zachary Van Winkle

**Affiliations:** Department of Sociology, Trinity College Dublin, Dublin, Ireland; Department of Social Sciences, Humboldt-Universität zu Berlin, Berlin, Germany; Centre for Research on Social InequalitieS (CRIS), Sciences Po, Paris, France; Nuffield College, University of Oxford, Oxford, UK

**Keywords:** Cross-country, Economic status, Family structure, Longitudinal methods

## Abstract

**Objectives:**

Widowhood has been shown to decrease surviving spouses’ economic well-being. However, previous research has focused mostly on income-related outcomes, and has been less attentive to the importance of wealth, the processual nature of spousal death, and cross-national variation. In this study, we assessed how total, housing, and nonhousing wealth changes over the process of widowhood across 11 European countries.

**Methods:**

Individual fixed-effects regressions and longitudinal data from the Survey of Health, Ageing and Retirement in Europe were used to estimate how household net total wealth, housing wealth, and nonhousing wealth changed 3 years prior and 6 or more years after spousal death relative to 4 or more years prior to widowhood in 11 European countries.

**Results:**

In all countries, household net wealth stayed relatively constant across the widowhood process, except for Austria, the Czech Republic, and Poland, where wealth declines were observed especially in the years following death. However, we found declines in housing wealth over the widowhood process, including prior to spousal death, across most countries in our sample, particularly in Austria, France, Denmark, the Czech Republic, and Poland. Declines in housing wealth were generally not reflected by changes in nonhousing wealth but coincided with leaving homeownership and downsizing.

**Discussion:**

Widowhood is associated with lower wealth, especially housing wealth, even in the years before spousal loss. Future research should focus on adjudicating the mechanisms behind country differences and exploring the implications of lost wealth following widowhood for surviving spouses’ well-being and intergenerational transfers.

While marriage—particularly long-lasting, stable marriage—has been associated with a range of economic benefits, marital dissolution has been linked to financial hardship (e.g., Bíró, 2013; Holden & Smock, 1991; Kapelle & Vidal, 2022; Kreyenfeld & Trappe, 2020; Raley & Sweeney, 2020). Divorce remains the most common route out of marriage, although most marriages in older age end with the death of one spouse (Carr & Utz, 2020). Widowhood and its consequences for older adults’ economic well-being have gained academic and public attention in recent years as old-age poverty rates continue to rise (Ebbinghaus, 2021; Kuitto et al., 2023).

This paper contributes to the existing literature on the association between widowhood and economic well-being in three ways. First, while a substantial body of research has focused on the link between widowhood and income measures (e.g., Gillen & Kim, 2009; Haveman et al., 2003; Sevak, 2004; Weir et al., 2002), the consequences of widowhood for the household’s wealth have attracted less attention. The small body of wealth research mostly focused on the United States and found mixed results on the association between widowhood and wealth (Goda & Streeter, 2021; Hurd & Wise, 1987; Poterba & Venti, 2017). Understanding the link between widowhood and household wealth more thoroughly is critical as sufficient wealth provides an important safety net with different wealth components providing specific advantages (Keister, 2000; Spilerman, 2000). While financial, nonhousing wealth (e.g., money in savings accounts, stocks, bonds) can easily be accessed and used to support current and future consumption even in the absence of income, housing wealth (i.e., real estate) can also be beneficial without being “consumed” (Keister, 2000). In contemporary aging societies, widowhood occurs mostly in older age when individuals are no longer active in the labor market (see Zueras et al., 2020). As seen in Table 1, European women and men experience widowhood, on average, in their 70s or later. Thus, income levels may be comparatively low due to the lack of labor market earnings while reliance on accumulated wealth—to support consumption and secure housing for all household members—can be expected to be high. The question of whether widowhood substantially reduces overall net wealth of the household or specific wealth components is thus particularly critical for the immediate and long-term financial standing of widowed households because it is unlikely that potential widowhood-induced wealth declines can be recovered in older age.

**Table 1.** Descriptive Statistics

Variable	Austria	Germany	Sweden	Spain	Italy	France	Denmark	Switzerland	Belgium	Czech Republic	Poland
	Mean (*SE*)	Mean (*SE*)	Mean (*SE*)	Mean (*SE*)	Mean (*SE*)	Mean (*SE*)	Mean (*SE*)	Mean (*SE*)	Mean (*SE*)	Mean (*SE*)	Mean (*SE*)
Widowhood sample											
Net wealth in €10,000	14.69 (19.92)	19.79 (27.11)	21.91 (30.91)	22.43 (30.22)	22.18 (28.10)	29.40 (37.37)	22.46 (31.85)	37.74 (62.18)	29.01 (29.00)	11.04 (16.48)	6.10 (7.48)
Net housing wealth in €10,000	12.18 (18.81)	14.76 (22.44)	14.57 (21.45)	21.06 (29.34)	19.96 (24.07)	22.52 (24.10)	12.83 (16.07)	26.08 (52.30)	20.19 (18.37)	9.41 (12.35)	5.76 (7.29)
Net nonhousing wealth in €10,000	2.51 (4.76)	5.04 (11.64)	7.34 (16.57)	1.37 (4.45)	2.22 (10.39)	6.89 (26.75)	9.63 (22.35)	11.66 (26.50)	8.82 (17.36)	1.62 (10.21)	0.34 (0.99)
Homeowner	0.49	0.56	0.73	0.92	0.82	0.80	0.70	0.57	0.76	0.68	0.72
Number of rooms in dwelling	3.68 (1.59)	4.13 (1.68)	4.01 (1.43)	4.19 (1.33)	3.70 (1.40)	4.54 (1.44)	4.29 (1.55)	4.40 (1.53)	4.67 (1.68)	3.26 (1.25)	2.64 (1.19)
Women	0.74	0.65	0.70	0.78	0.77	0.73	0.66	0.67	0.68	0.77	0.72
Age	72.11 (8.73)	70.94 (9.13)	73.97 (8.71)	75.26 (8.82)	72.60 (8.44)	72.40 (9.69)	72.39 (9.32)	73.98 (8.62)	73.54 (9.29)	71.25 (7.79)	71.06 (8.90)
Age at widowhood	73.07 (8.27)	71.97 (8.85)	74.84 (8.32)	76.15 (8.50)	74.03 (7.86)	73.47 (9.64)	73.17 (9.10)	74.76 (7.93)	74.57 (8.95)	72.01 (7.46)	71.22 (8.71)
Observations	901	1,198	1,412	1,992	1,603	1,396	1,202	902	1,439	1,442	915
Individuals	231	302	323	483	346	330	273	205	312	373	235
Continuously married sample											
Net wealth in €10,000	23.54 (31.55)	27.93 (34.36)	33.29 (37.19)	28.06 (36.74)	27.40 (29.80)	36.72 (40.56)	36.56 (41.53)	55.90 (79.42)	42.42 (40.65)	15.35 (15.29)	8.87 (9.51)
Net housing wealth in €10,000	19.15 (27.18)	20.00 (26.75)	20.77 (23.57)	24.43 (32.86)	23.80 (23.91)	28.37 (26.19)	18.95 (18.80)	36.74 (63.49)	28.51 (22.56)	12.61 (12.61)	8.08 (8.72)
Net nonhousing wealth in €10,000	4.39 (11.64)	7.93 (15.58)	12.52 (22.04)	3.63 (12.74)	3.61 (13.63)	8.35 (28.16)	17.61 (31.49)	19.16 (35.15)	13.91 (27.92)	2.74 (6.41)	0.79 (2.50)
Homeowner	0.64	0.72	0.84	0.94	0.87	0.87	0.89	0.72	0.89	0.83	0.80
Number of rooms in dwelling	4.17 (1.55)	4.51 (1.58)	4.57 (1.45)	4.23 (1.31)	3.90 (1.35)	4.80 (1.41)	4.86 (1.51)	4.97 (1.47)	5.11 (1.62)	3.58 (1.39)	3.01 (1.37)
Women	0.48	0.49	0.49	0.49	0.49	0.48	0.49	0.48	0.49	0.49	0.50
Age	66.38 (8.68)	65.93 (8.80)	68.22 (8.71)	67.61 (9.54)	66.68 (8.87)	65.73 (9.28)	64.86 (8.91)	66.45 (8.98)	65.39 (9.30)	66.36 (8.28)	66.05 (8.43)
Observations	9,039	12,271	11,907	15,004	14,399	12,405	10,123	8,503	14,983	11,524	4,336
Individuals	2,819	3,933	3,424	4,686	4,022	3,566	2,794	2,281	4,247	3,447	1,239

*Notes*: *SE* = standard error; SHARE = Survey of Health, Ageing and Retirement in Europe. Data are from the SHARE survey (release 8.0.0). Net wealth was converted to constant 2015 German Euros using the Consumer Price Index and then top- and bottom-coded wealth at the 0.1% level.

Second, early research on the financial consequences of widowhood has commonly conceptualized spousal death as a single point-in-time event (e.g., Hurd & Wise, 1987), despite Carr and Utz’ (2001) call for bereavement to be conceptualized as a process. Indeed, rather than a static status, the process of spousal death—just like marital dissolution through separation and divorce (Kapelle & Baxter, 2021)—often includes anticipation prior to widowhood, a phase of intensive grief at widowhood and thereafter, and potential adaptation in later years. This dynamic nature of widowhood may also have relevant implications for the financial well-being of surviving spouses and their households more generally. In the present study, we thus take a longitudinal approach that acknowledges the processual nature of widowhood and concurrent dynamic changes in household wealth.

Finally, the consequences of divorce for income have been assessed across different country contexts with research highlighting substantial cross-country variation (e.g., Van Winkle & Leopold, 2021). In contrast, little is known about whether widowhood and its consequences for economic well-being, specifically wealth, are experienced differently across different social welfare contexts. However, numerous country-specific aspects including the availability and generosity of bereavement support payments or regulations around inheritance likely influence the wealth consequences of widowhood.

We address the mentioned shortcomings in previous research and assess three research questions. First, *how does total household net wealth change across the process of widowhood?* Second, *does the process of widowhood affect wealth components, specifically housing and nonhousing wealth, differently?* Third, *how do widowhood-related wealth changes differ across European countries?* Focusing on household net wealth, our study provides a thorough picture of the financial situation of the household throughout widowhood. Note that we do not focus on intraspousal dynamics, such as transfers before spousal death (*inter vivos*) or inheritances after spousal death. To address our three research questions, we use individual fixed-effects regression models and longitudinal data on European countries from the Survey of Health, Ageing and Retirement in Europe (SHARE). Specifically, we draw on data from a diverse set of Eastern European (Czechia and Poland), Southern European (Italy and Spain), Western European (Austria, Belgium, France, Germany, and Switzerland), and Nordic (Sweden and Denmark) countries.

## Background

### Wealth and Its Components in Older Age

Although access to sufficient wealth is central to the economic well-being across the life course, it has particularly high relevance in older age (Keister, 2000; Spilerman, 2000). Especially following retirement, labor market income ceases, and wealth is vital to finance aspects of everyday life and maintain desired living standards (Killewald et al., 2017). As such, the anticipation of retirement is one of the most central savings motives throughout the life course (Keister, 2000; Modigliani, 1986). Older adulthood is therefore a period where wealth tends to be consumed and is no longer accumulated further (Spilerman, 2000). In that regard, wealth shocks during older age, for example, resulting from marital dissolution, can have critical implications for the economic standing of surviving spouses but also their adult children.

Wealth is not singular but is comprised of various components, including, for instance, real estate, business ownership, stocks and bonds, savings, retirement accounts, life insurance, jewelry, cars, and collectables (Spilerman, 2000; Wolff & Zacharias, 2009). Among the most important is housing wealth. For many older homeowners, their primary residence makes up by far the largest share of their wealth portfolio (Hurd & Wise, 1987). Nonhousing wealth consists of not only financial assets but also the value of cars and businesses. Savings are the most common form of financial asset. However, many older adults also have investments in the form of stocks, bonds, and other financial instruments (Hurd & Wise, 1987). While nonhousing wealth, specifically financial assets, can be easily liquidized to cover expected and unexpected costs, housing wealth has a function of its own and its liquidation is costly.

### Wealth Across the Widowhood Process

A long line of research has assessed the impact of widowhood on individual well-being through a life course lens, where spousal death is a stressful life events that induce change and require adjustments from surviving spouses (see Lin & Brown, 2020). Compared to other life events, widowhood is thought to have mostly negative consequences due to the undesirability of spousal loss, even if it is often a predictable life event in older age (Dohrenwend & Dohrenwend, 1974; Thoits, 1983). However, the severity and persistence of the negative consequences of widowhood are thought to vary according to individual vulnerability, such as coping resources or strategies, dispositions, or social location, as well as the history of the marital relationship (Kessler, 1979; Pearlin et al., 1981; Wheaton, 1990).

**Figure 1.**
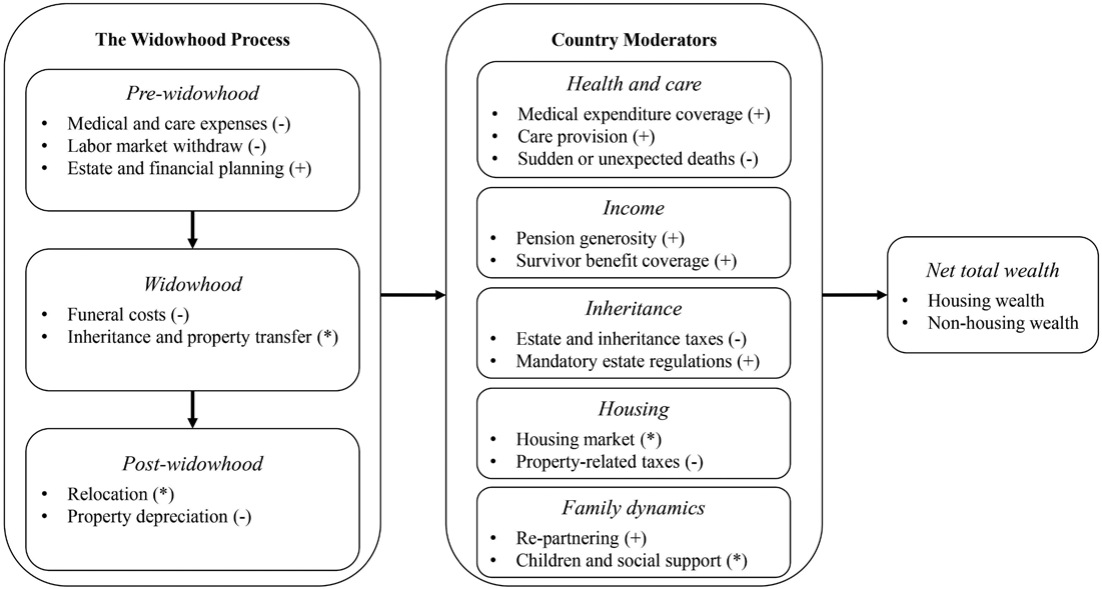
Conceptual overview of individual- and couple-level factors and country moderators on wealth across the widowhood process.

Longitudinal research on the economic consequences of widowhood commonly assesses pre- and post-widowhood phases. However, we follow Carr and Utz (2001) and conceptualize spousal death as a process that occurs over a prolonged time span rather than a point-in-time dichotomous event. Similar approaches have been taken to assess wealth and marital dissolution through separation and divorce (Kapelle & Baxter, 2021). In line with those approaches, widowhood can also be considered a process consisting of a pre-widowhood anticipation phase, the immediate time of widowhood, and the post-widowhood phase. Although this is not an exhaustive list, Figure 1 shows the main factors linking bereavement and wealth throughout this widowhood process. Note that we associate individual- and couple-level factors with the point in the widowhood process at which they may be particularly relevant to wealth. However, spillover and feedback effects mean that individual-, couple-, and country-level factors that are relevant for wealth early in the widowhood process may remain relevant later.

In aging societies where widowhood commonly occurs in older age, spousal death is often preceded by long-term sickness or declining health and is therefore anticipated by the surviving spouse with potentially important implications for wealth. During the pre-widowhood phase, medical and care expenses, premature withdraw from the labor market, and pre-bereavement estate and financial planning are important factors that may affect wealth (see Figure 1). Many of the leading causes of death in Western countries, such as cardiovascular disease, cancer, and dementia, not only entail protracted medical treatment but also long-term care (e.g., Teggi, 2020). This can cause considerable financial strain during the pre-widowhood period. If dying spouses still work for pay, illness may cause reduced work hours and an earlier withdrawal from the labor market. Moreover, if surviving spouses perform caregiving duties, further costs may be incurred by forgone labor income. This could drive couples to consume wealth holdings prematurely to cover financial gaps or maintain their standard of living. The expectation of spousal death may additionally prompt those without wills to discuss financial matters and plan estates, which has been shown to be associated with higher wealth retention among surviving spouses (Choi & Carr, 2023). Hence, household wealth levels—particularly of nonhousing wealth, which can more easily be accessed—may decline in years before widowhood.

During the widowhood phase, funeral costs are thought to be linked with wealth consumption, but inheritances and property transfers may be associated with increased wealth directly following spousal death (see Figure 1). Substantial costs that are commonly covered by surviving spouses are incurred immediately following bereavement (Fan & Zick, 2004, 2006). These costs include, for instance, expenses for the death certificate and other doctor’s services, funeral director’s services, burial or cremation, minister or celebrant, and additional third-party services (e.g., flowers, catering, venue hire, limousine hire). According to SunLife (2023), a UK-based financial services company, the average cost of dying is around 10% of an average salary. Most families have to draw on savings, sell belongings, or borrow money from friends, relatives, or loan providers to cover funeral costs. Thus, funeral costs in the year of widowhood can substantially drain a household’s wealth.

While funeral costs are commonly covered within the year of widowhood, bereavement may negatively affect wealth in the years after spousal death. In the post-widowhood period, factors related to relocation and property depreciation may be particularly relevant (see Figure 1). Marital dissolution in the form of divorce or bereavement leads to a decrease in economies of scale as household costs are no longer divided by both spouses. Thus, per capita expenses within the household increase, which tend not to be completely offset by survivor pensions or life insurance policies (Gillen & Kim, 2009). In addition, surviving spouses may relocate in the years after bereavement, for example, to reside near living kin, reduce per capita costs, or downsize to accommodation that is more manageable (Bonnet et al., 2010; Zilincikova et al., 2023). Although relocation may lead to lower costs over time, it is initially associated with a wide range of costs. For homeowners, downsizing means the sale of their current property and acquisition of a smaller property (Herbers et al., 2014; Keese, 2012). This would ultimately result in a loss of housing wealth but may lead to an increase in financial wealth depending on transaction costs.

### Country Differences in Widowhood and Wealth

Across the process of widowhood, numerous institutional and cultural differences between countries may generate cross-national variation in the link between spousal death and wealth. An overview of country-level moderators that may affect the link between individual- and couple-level factors and wealth across the widowhood process is displayed in Figure 1. An overview of how these country-level moderators vary across our study countries is displayed in Table 2.

**Table 2.** Indicative Summary of Country Moderators for the Consequences of the Widowhood Process on Wealth

Country	Health and care			Internment costs		Inheritance		Housing		Income			Family	
	Medical expense^a^	Unmet care needs^b^	Unexpected death^c^	Cost of dying^d^	Funeral benefit^e^	Tax exemption threshold^f^	Default share^g^	Ownership^h^	Property tax^i^	Pension replacement rate^j^	Survivor benefit level^k^	Life insurance^l^	Support from children^m^	Repartnering^n^
Austria	4.0	44.1	25.3	7.1	0.0	—	33	54.2	0.03	74.1	54.8	1.7	12.9	6.2
Belgium	4.1	39.2	29.6	9.1	2.0	17,133	Usufruct	71.3	0.67	43.5	88.6	2.8	9.6	7.4
Czech Republic	2.7	70.3	25.1	3.6	0.1	—	Divided	78.3	0.10	47.4	63.3	0.8	28.2	8.8
Denmark	3.0	37.2	26.7	2.4	6.6	Exempt	50	59.2	0.64	73.1	22.5	7.7	16.4	8.7
France	2.2	42.0	23.9	10.1	0.1	Exempt	25	64.7	0.85	57.6	48.5	5.8	8.1	5.6
Germany	3.2	40.7	21.3	16.4	3.1	571,098	50	49.1	0.11	43.9	68.6	2.4	13.5	11.1
Italy	3.6	56.1	25.2	5.7	—	1,142,197	Divided	73.7	0.58	76.1	53.7	4.9	7.0	2.8
Poland	2.3	70.0	26.8	2.3	0.0	Exempt	25	86.8	1.06	29.3	104.8	0.7	9.7	4.6
Spain	4.0	41.6	29.0	8.7	0.2	18,226	50	75.8	0.62	80.4	53.6	1.8	8.3	5.1
Sweden	3.4	48.1	26.3	6.9	—	—	100	64.9	0.43	62.3	28.3	9.1	11.5	7.2
Switzerland	5.5	44.5	33.2	—	0.0	Exempt	50	42.2	—	39.9	30.1	3.0	6.5	8.3

*Notes*: SHARE = Survey of Health, Ageing and Retirement in Europe. “—” indicates that no information was available for the specific indicator for that country.

^a^Out-of-pocket spending as share of final household consumption, 2021 (or nearest year). See OECD (2023a).

^b^Unmet long-term care needs among people aged 65 and older living at home, 2019–2020, with at least one Activities of Daily Living (ADL)/Instrumental Activities of Daily Living (IADL) limitation in percent. See OECD (2023a).

^c^Estimated share of deaths with less than 1 month of sickness. Own calculations based on SHARE exit interviews.

^d^Average cost of a typical end-of-life ceremony, such as the cost of burials, cremations, and funerals, as a percent of average salaries in 2020. See SunLife (2023).

^e^Per capita survivor benefits in kind for funeral expenses in 2019, or nearest date, at constant prices and Purchasing Power Parity (2015) in USD. See OECD Social Expenditure Database.

^f^Tax exemption threshold for donor’s spouse in 2020 USD. See OECD (2021).

^g^Spousal inheritance policies absent a written will for married spouses with children. Percentages indicate the share that the surviving spouse keeps in case the couple had children. Divided denotes that wealth is divided equally with children. Usufruct denotes the right to use (housing) wealth without any default minimum ownership. See Nicholas and Baum (2019).

^h^Eurostat estimate of share of people living in households owning or renting their home, 2021.

^i^Implicit recurring property tax in percent in 2021 or the nearest date. See Barrios and colleagues (2019).

^j^Gross pension replacement rate for men’s average earnings through mandatory schemes. See OECD (2023c).

^k^Average level of survivor pensions relative to old-age pensions in 2016 or the nearest date. See OECD (2018).

^l^The life insurance penetration rate is calculated as the ratio of total insurance premiums to gross domestic product in the year 2022. See OECD (2023b).

^m^Estimated share of SHARE respondents who receive help from children living outside the household based on own calculations.

^n^Estimate share of SHARE widows and widowers who are observed to repartner based on own calculations.

For example, during the pre-widowhood phase, national healthcare and elderly care systems will likely affect the extent that to-be widows and widowers need to draw on wealth (see Figure 1). In countries with well-developed public healthcare systems, spouses will be less likely to be confronted with out-of-pocket medical expenses. Across Europe, the proportion of older adults’ out-of-pocket medical expenses comprises 5.5% of household consumption in Switzerland, but only 2.2% in Poland. In addition, spouses will be less likely to draw on wealth to pay for care work or withdraw from the labor market to perform care work themselves in countries with developed long-term care systems. In Denmark only 37% of older adults have unmet care needs compared to 70% in the Czech Republic and Poland. Relatedly, medical and care expenses will be less relevant in countries where sudden deaths without prolonged pre-death phases are more common, such as in Switzerland, where 33% of surviving spouses report pre-death being preceded by no or just a brief phase of illness.

The costs of dying and inheritance regulations, and therefore the impact those aspects have on the association between widowhood and wealth, vary drastically across Europe (see Figure 1). SunLife (2023) estimated the costs associated with death to be highest in Germany (16.4% of an average salary) and lowest in Denmark and Poland (2.4% and 2.3% of an average salary, respectively). To cover these costs, some countries may offer governmental support in the form of funeral expenses payments or require individuals to have some form of life insurance. Aside from funeral costs, countries with high inheritance tax rates or default inheritance regulations that benefit children over spouses may exacerbate the impact of the widowhood phase on wealth. This is particularly the case in Belgium and Poland, for example.

In the post-widowhood phase, country differences in housing markets, pension and survivor benefit schemes, and family dynamics may prove to be particularly important (see Figure 1). Widows and widowers may profit less from homeownership in countries with relatively high property taxes, such as Poland or France, compared to countries such as Austria with low property taxes. The prime strategy for countries to protect the wealth holdings of widows and widowers in the years following spousal death is to replace lost household income via survivor pensions or benefits (James, 2009). However, countries differ to a large extent in both the reach and generosity of payments to surviving spouses. While survivor pensions are relatively uncommon in Norway, with under five recipients per 100 old-age pension recipients, and are only equal to 10% of mean old-age pensions, survivor pension recipiency is six times higher in Germany where survivor pensions are close to 60% of mean old-age pensions (OECD, 2018). In countries where payments are means-tested and meager, surviving spouses will be more likely to consume wealth to secure their standard of living. Adult children may help their surviving parent and mitigate the need to draw on wealth, for example, through cash transfers. Support from adult children to elderly parents is particularly common in the Czech Republic, where 28% of older adults receive some financial support, compared to countries, such as Switzerland with just 6.5%.

### Previous Research

Previous longitudinal studies on widowhood and surviving spouses’ economic well-being compared only two time points (e.g., Angel et al., 2007; Haveman et al., 2003; Hungerford, 2001; Morgan, 1981; Sevak, 2004), which reflects the conceptualization of widowhood as a two-stage process in previous research (i.e., being married—and before widowhood—compared to being widowed). Additionally, research commonly focused on income measures rather than wealth. Results generally pointed to a decrease in economic well-being in the year of spousal death. However, results were mixed as to whether the economic well-being of widows and widowers improves or remains stable in the years following death.

Few studies moved beyond standard measures of economic well-being, for example, household income, to study the impact of widowhood on wealth. Poterba and Venti (2017) explored the role of health shocks and spousal loss in contributing to the drawdown of retirement wealth in the United States. Bereavement was associated with a drop in net wealth, but affected retirement financial assets, housing equity, and vehicles most (see also Hurd & Wise, 1987). Goda and Streeter (2021) in contrast showed increases in wealth following widowhood in the United States; however, they conceded that their result was largely driven by adjusting for household size.

Previous research on widowhood and economic well-being, and specifically wealth, has tended to focus on the United States. In a rare exception, Nicholas and Baum (2019) pooled data from the United States and Europe to demonstrate that the use of wills and testimonies is associated with greater retained wealth among surviving spouses. In sum, the current study goes substantially beyond the current state of the art to assess country differences in total wealth and its components across the process of widowhood.

## Data and Methods

To examine the association between widowhood and wealth across Europe, we used longitudinal data from the SHARE. SHARE is a multidisciplinary and cross-national panel database of micro data of individuals aged 50 or older (see Börsch-Supan et al., 2013). Since 2004, SHARE data have been collected annually using computer-assisted personal interviews with currently eight waves of data available. For Wave 8, which took place during the coronavirus disease 2019 (COVID-19) pandemic, interviews were conducted using computer-assisted telephone interviews. To assess whether this mode change affected our results, we ran analyses both with and without Wave 8. As the results did not differ substantially, we included Wave 8 in our main analyses. See Supplementary Figures 1 and 2 for results that exclude Wave 8.

Whereas 11 European countries and Israel were included in the first SHARE wave, other countries have been added over time, resulting in data for 29 European countries and Israel in Wave 8. Because not all countries were included in all waves resulting in some countries having to limited longitudinal data that prevents analyses of changes over time, we used data from 11 European countries with sufficient longitudinal data and sample sizes for the present study: Austria, Belgium, Czech Republic, Denmark, France, Germany, Italy, Poland, Spain, Sweden, and Switzerland.

SHARE data cover a range of topic areas including socioeconomic status, health, and social and family networks. The regular panel modules included in Waves 1, 2, 4, 5, 6, 7, and 8 predominantly cover respondents’ current situation. To gain an understanding of respondents’ earlier living conditions or life course events and transitions, data on respondents’ life histories were collected as the SHARELIFE component in Wave 3 and, for respondents that did not complete the survey in Wave 3, in Wave 7.

SHARE data were particularly relevant for the current study as they (a) provide information on relevant measures including marital status as well as current financial and real assets and debt, (b) contain sufficiently large country-specific samples for a cross-country comparative analytical approach, and (c) guarantee a high level of comparability between countries. Data for the current study stemmed from the regular panel modules on respondents’ current situation while we excluded retrospective SHARELIFE data.

In our analyses, we used data that were edited and imputed by the SHARE survey team. Although imputed data were provided for the majority of relevant variables, some variables, such as the number of rooms in the dwelling—which we used for supplementary analyses—were not imputed. To handle missing data in these variables, we built on SHARE imputations and imputed missing data with chained equations using Stata’s *mi* procedure (Version 17). To enhance the quality of our imputations, a range of relevant auxiliary variables, such as respondents’ and their partner’s education, or the number of children and grandchildren, were included. A detailed list of the entire set of variables used in the imputation process, including all auxiliary variables, can be found in Supplementary Table 1. The table additionally provides the share of missing values addressed through our own or SHARE-provided imputations. Estimation results from five imputed data sets were combined using Rubin’s rules (Rubin, 1987).

For the analytical sample, successfully interviewed individuals aged 50 years and older living in private households were selected if they were either continuously married or if they experienced the death of their spouse during their panel participation. Due to our analytical approach, we could only include respondents who took part in at least two waves. We base our analyses on unbalanced samples with 33% of respondents observed twice, 29% observed three times, 16% observed four times, 7% observed five times, 9% observed six times, and 6% observed over all available seven waves. Hence, our analyses are affected by panel attrition, as is a common issue with longitudinal analyses. To avoid further bias, we use imputed data. In total, the final analytical sample comprised 39,871 respondents with 138,896 individual-year observations. We observed 3,413 widowhood transitions. The breakdown of our sample across the countries can be found in Table 3.

**Table 3.** Number of Individuals, Individual-Year Observations, and Transitions

Country	Observations (*n* = 138,896)	Individuals (*n* = 39,871)	Widowhood transitions	
			Overall (*n* = 3,413)	Women (*n* = 2,432)
Austria	9,940	3,050	231	167
Germany	13,469	4,235	302	198
Sweden	13,319	3,747	323	216
Spain	16,996	5,169	483	372
Italy	16,002	4,368	346	264
France	13,801	3,896	330	243
Denmark	11,325	3,067	273	178
Switzerland	9,405	2,486	205	138
Belgium	16,422	4,559	312	207
Czech Republic	12,966	3,820	373	284
Poland	5,251	1,474	235	165

*Notes*: SHARE = Survey of Health, Ageing and Retirement in Europe. Data are from the SHARE survey (release 8.0.0).

### Measures

Our main outcome variable was *household net wealth*, which is defined as the sum of real and financial assets minus debts. Financial assets reflect the sum of values of accounts, bonds, stocks, mutual funds, and savings. Real assets pertain to the value of primary residence net of mortgage, other real estate, owned businesses, and owned cars. A detailed description of how wealth data were collected in SHARE is provided in Supplementary Material. We converted net wealth to constant 2015 German Euros using the Consumer Price Index and then top- and bottom-coded wealth at the 0.1% level.

In addition to net wealth, we were also interested in changes in certain wealth components. We thus disaggregated the overall net wealth measure into housing net wealth and nonhousing net wealth. Whereas housing net wealth referred to the real estate property including potential mortgage debt, nonhousing net wealth referred to the remaining more liquid resources (Spilerman, 2000).

Just like income data, wealth data are highly skewed, but common transformations such as the natural log are not suitable as net wealth measures contain zero and negative values that would be dropped by a log transformation (Killewald et al., 2017). To deal with the skewness of the data while maintaining zero and negative values, we applied an inverse-hyperbolic sine (ihs) transformation (e.g., Friedline et al., 2015; Killewald et al., 2017).

The main explanatory variable was a categorical indicator of the time before and since widowhood in six categories: (1) married and at least 4 years prior to widowhood (reference), (2) married and between 2 and 3 years prior to widowhood, (3) married and 1 year prior to widowhood, (4) the year of widowhood, (5) 1 year after widowhood, (6) 2–3 years after widowhood, (7) 4–6 years after widowhood, and (8) more than 6 years after widowhood. Cell sizes across those categories and by country are provided in Supplementary Tables 2 and 3.

We estimated our fixed-effects regression models with a small set of time-variant control variables: Respondents’ age as a categorical variable (50–59 [Ref], 60–69, 70–79, 80–90) was added to capture maturation effects. Additionally, we added a dummy to flag imputed data to account for potential systematic differences between imputed and nonimputed data. Finally, we also include a dummy to flag Wave 8 to account for the COVID-19 pandemic and associated changes in the interviews. As the association between widowhood and net wealth can be expected to work partially through mechanisms such as repartnering, living arrangements (e.g., living with family or friends, children in the household) or family support, as well as employment, we decided against accounting for those potentially mediating factors in our main analyses and keep our regression models parsimonious. To conduct a first assessment of whether these factors may be linked to our association of interest, we conducted supplementary analyses that included dummies for repartnering and being in employment as well as a continuous measure for the household size. Results are presented in Supplementary Figures 3 and 4 and are in line with our main results. Future research should, however, more appropriately address such underlying mechanisms.

### Analytical Strategy

To analyze the association between widowhood and net wealth, we used multivariate regression analyses using a fixed-effects regression approach. To ease the readability of the results, coefficients are presented in graphical format. Fixed-effects regression models leveraged the panel data and made exclusive use of the within-individual variation in the explanatory and outcome variables over time (Allison, 2009). This approach more appropriately addressed selection effects. Thus, time-invariant observed or unobserved factors (e.g., family background, gender, or ethnicity) did not bias our fixed-effects analyses, thereby reducing omitted variable bias. For all regression models, standard errors were corrected for clustering of observations within individuals. The replication code is available at https://osf.io/gyc7f/.

## Results

### Sample Description

Summary statistics of our key variables for our sample of widowed and continually married respondents are displayed in Table 1. As would be expected, we found large differences in household net wealth across countries. However, in all countries net wealth holdings were smaller among our widowed compared to our married sample. Swiss households tended to have the most household net wealth (widowed sample: 378,300 Euros; continuously married sample: 561,500 Euros), while Polish households had the least wealth (widowed sample: 61,000 Euros; continuously married sample: 88,700 Euros) among our country sample.

We found substantial differences between the widowed and continuously married sample across all countries for both housing and nonhousing wealth. However, across all countries, households held on average substantially more wealth in the form of housing wealth compared to nonhousing wealth. For example, Austrian households in our widowed sample owned on average 121,800 Euros in housing wealth and 25,100 Euros in nonhousing wealth, compared to 191,500 Euros and 43,900 Euros, respectively, for continuously married Austrian households.

In sum, the descriptive results suggested that widowhood is associated with declines in both housing and nonhousing wealth, despite large cross-national differences. However, our widowed sample comprised more women than men and was slightly older compared to the sample of continuously married older adults. Those and other differences between both groups could be generating the differences we observed.

### Net Wealth Changes Throughout the Widowhood Process

**Figure 2.**
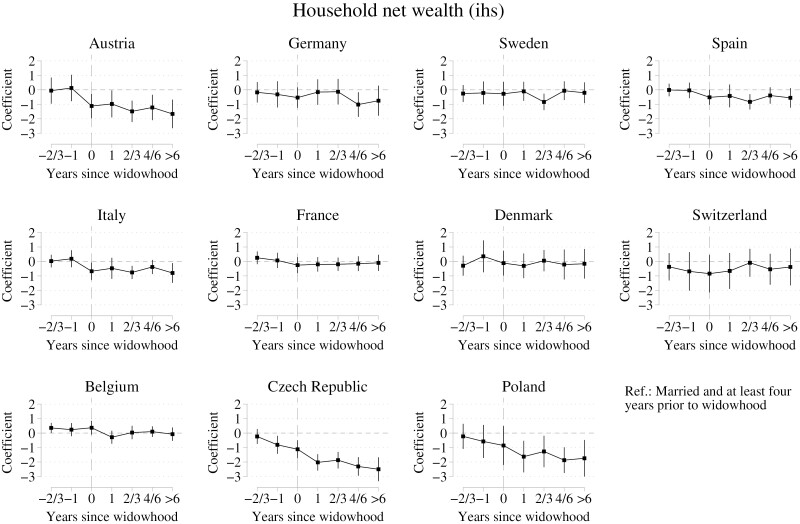
Fixed-effects regression coefficients for household net wealth (ihs-transformed) through widowhood across 11 countries. Reference “married and at least 4 years prior to widowhood.” Whiskers indicate 95% confidence intervals. Data are from the SHARE release 8.0.0 (Waves 1–8; unweighted; multiply imputed). SHARE = Survey of Health, Ageing and Retirement in Europe.

Regression results from individual fixed-effects regressions of ihs-transformed household net wealth on time to and since widowhood across 11 European countries are displayed in Figure 2. In the majority of our study countries, household net wealth stayed relatively constant across the widowhood process. The lack of change in net wealth before, during, and after widowhood was particularly evident in Denmark, Sweden, Spain, Italy, and France.

In a small number of countries, we observed declines in household net wealth across the widowhood process to varying degrees. For example, in the Czech Republic, wealth began to decline well before the year of spousal death, highlighting anticipation effects. Czech widows and widowers already had around 55% less wealth 1 year prior to spousal loss compared to 4 or more years prior to widowhood. Additionally, we observed statistically significant wealth declines in the year of widowhood for Austria, Italy, and the Czech Republic. Substantial—although statistically nonsignificant—declines in the year of widowhood were also found in Poland. Declines vary across those countries. For example, widows and widowers in Austria had roughly 1.1 ihs-transformed or 78% less net wealth in the year of widowhood compared to 4 or more years prior to widowhood, while wealth declined by 0.7 ihs-transformed wealth or 50% in Italy. For three countries, Poland, the Czech Republic, and Austria, we additionally found further, critical declines in years after widowhood.

### Changes in Wealth Components: Housing Wealth Compared to Nonhousing Wealth

**Figure 3.**
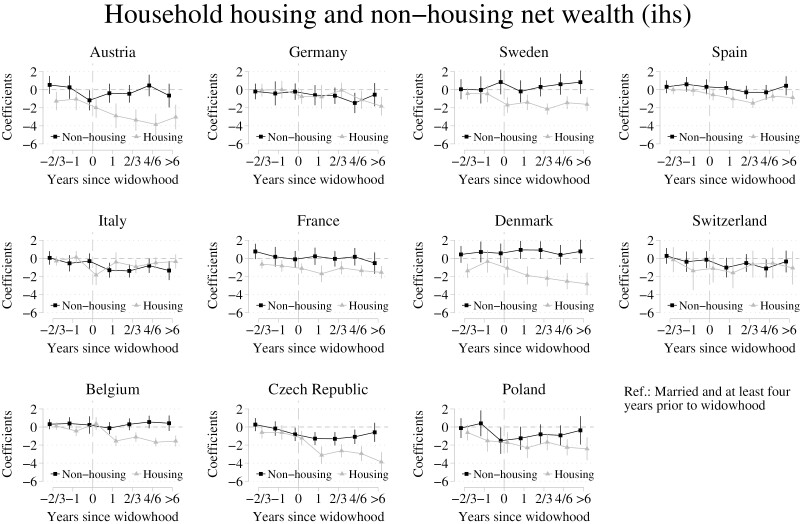
Fixed-effects regression coefficients for household net housing and nonhousing wealth across 11 countries. Reference “married and at least 4 years prior to widowhood.” Whiskers indicate 95% confidence intervals. Data are from the SHARE release 8.0.0 (Waves 1–8; unweighted; multiply imputed). SHARE = Survey of Health, Ageing and Retirement in Europe.

Analyses that were conducted separately on housing and nonhousing wealth showed more country variation in the wealth consequences of the widowhood process than the aggregated analyses on total net wealth (see Figure 3). We found declines in housing wealth over the widowhood process across most countries in our sample. The largest housing wealth declines could be found in Austria, France, Denmark, the Czech Republic, and Poland. In many of these countries, housing wealth declined sharply in the pre-widowhood phase already. For example, in Austria housing wealth declined by nearly 72% 2–3 years prior to death, and declined further to 94% less 1 year and 98% 3–6 years after death. Most noticeable for Austria, Denmark, and the Czech Republic, housing wealth declined further in years after widowhood.

In contrast, we found that in most countries nonhousing wealth fluctuated only to a small degree. This suggests that housing wealth was not on average being translated into nonhousing wealth. Only in Denmark, a country where housing wealth declined sharply relative to 4 or more years prior to spousal death, was nonhousing wealth roughly 50% higher in the widowhood and early post-widowhood period. Otherwise, we found some evidence that nonhousing wealth decreased in the widowhood and post-widowhood periods in the Czech Republic and Poland, as well as in Italy and Switzerland to a smaller degree. For example, nonhousing wealth in the Czech Republic was roughly 71% less 1 year after spousal death compared to 4 or more years prior to bereavement.

Linking our regression results to Table 2, we can identify no obvious correlations between the outlined indicators and wealth declines or lack of declines, highlighting that underlying mechanisms are likely complex. For example, across the three countries that see the largest housing wealth declines, the surviving spouse inherits 33% of the deceased partner’s wealth in Austria if children are present, in the Czech Republic the deceased person’s wealth is divided equally with children, and in Poland the surviving spouse inherits 25% if children are present. Similar regulations can be found in France and Italy where (housing) wealth, however, does not decline to similar degrees.

### Supplementary and Robustness Analyses

We conducted four additional supplementary and robustness analyses. First, we delved deeper into the relationship between the widowhood process and housing wealth. This was important because we found that changes in wealth over the process of widowhood were generally concentrated among housing wealth. We therefore assessed the relationship between widowhood and homeownership on the one hand, and widowhood and the number of rooms on the other hand. Results of these analyses are presented in Supplementary Figures 5 and 6. We found that downsizing, reflected by the number of rooms available in the dwelling, could be observed in most but not all countries. Specifically, we observed substantial downsizing over time in Sweden, Denmark, and Switzerland, but only moderate and delayed downsizing in Spain, Germany, France, and Austria. This partially overlapped with a reduced likelihood for respondents to own the home in which they reside. We found considerable reductions in homeownership for Austria, Germany, Denmark, Belgium, Czech Republic, and Poland, but no or only marginal changes in Italy, Spain, France, Sweden, and Switzerland.

Second, we conducted analyses only on women, because previous research has found that the economic consequences of marital dissolution are generally more severe for women compared to men (e.g., Holden & Smock, 1991; Kapelle & Vidal, 2022). In addition, women are considerably more likely to become widowed than men because of women’s higher average life expectancy. However, we find no noteworthy differences between the results presented here and those for only women (see Supplementary Figures 7 and 8). This could be due to wealth belonging to the household, as opposed to individual labor or pension income.

Third, we assess the impact of using per capita wealth instead of household wealth on our results. This approach aligns with that of Goda and Streeter (2021), who reported an increase in wealth following widowhood. The per capita results exhibit a slight upward bias compared to our main findings, although the overall patterns remain consistent (see Supplementary Figures 9 and 10). Such an upward bias is anticipated because we halve the wealth during our reference period, that is, marriage. The per capita transformation inherently assumes that each spouse can access and benefit from only half of the total household net wealth during the marriage. We argue that this assumption is critical and potentially flawed, especially considering that most wealth is held in housing property—a component that benefits both spouses equally.

Finally, we also conducted analyses excluding the imputed data and found that the results reflect those presented here (see Supplementary Figures 11 and 12).

## Conclusion

In this study, we sought to address three research questions: First, how does household net wealth change across the process of widowhood? Second, does the process of widowhood affect wealth components differently? Third, how do widowhood-related wealth changes differ across European countries? We drew on individual fixed-effects regression models and longitudinal data to assess how total wealth as well as housing and nonhousing wealth changed over the widowhood process across 11 European countries. We found that although total household net wealth tended not to vary over the widowhood process for the majority of countries, housing wealth declined across most of our study countries, especially in the years following spousal death.

Although we found large cross-national differences in changes to wealth associated with widowhood, our results did not display any obvious country clusters. For example, housing wealth declined most in Austria, the Czech Republic, and Denmark, whereas it declined later and to a lesser degree in Germany, Poland, and Sweden. Therefore, we observed as much or more variance within than between common welfare state regime typologies. This suggests that existing typologies (e.g., Esping-Andersen, 1990) may be of little use in understanding country differences and similarities in how wealth changes with spousal death. Future research should continue to take a comparative approach but examine more fine-grained institutional and cultural factors than what we were able to do in this first cross-national assessment.

We found housing wealth is most sensitive to widowhood, but it was out of the scope of this study to delve into the mechanisms linking spousal death and housing wealth across countries. For example, does housing wealth decline following widowhood due to leaving homeownership, downsizing, or neglecting to maintain the property? Our supplemental results suggested that leaving homeownership may drive declines in housing wealth rather than downsizing. More systematic research is needed as to why downsizing was not correlated with declines in housing wealth across all countries. This could point to the importance of regional rather than national housing markets. For example, selling a larger residence in a rural area to buy an apartment in an urban center may be downsizing, but would unlikely eliminate housing wealth holdings.

It should be noted that losses in housing wealth did not directly translate into gains in nonhousing wealth. More research is needed to understand where reported housing wealth “goes” if there is no boost of nonhousing wealth (i.e., financial wealth) following a depletion of housing wealth. This could mean, for example, that real estate was sold under value, older adults underestimated their housing wealth, or that wealth was bequeathed directly to adult children. Passing wealth to adult children may not indicate a social problem, especially if surviving spouses have sufficient economic resources to secure their desired standard of living. However, it becomes a different matter if wealth is actually lost, for example, due to selling in an unfavorable market or if liquidized housing wealth is used to cover costs associated with partner loss. It becomes even more problematic if surviving spouses no longer have sufficient wealth or economic resources to cover their future needs, such as assisted living, care, medical costs, or residential maintenance.

It was out of the scope of this study to identify which social groups are at the greatest risk of losing considerable housing wealth following widowhood. An intuitive next step for research on widowhood and wealth should be to look at how the consequences of spousal loss vary across the wealth distribution in different European countries. Here it is important to note that small losses among those with little wealth may have large implications for surviving spouses’ ability to live their lives according to their own plan, but may also impede the transfer of wealth to poorer adult children who may profit the most from intergenerational transfers.

While more fine-grained research is needed to identify particularly disadvantaged groups and to understand the relevance of different mechanisms, the present study provides a first step toward a better understanding of how widowhood is linked to wealth changes over time and across different contexts. Our findings help us to highlight some relevant areas for policy debates. Amid increasingly competitive housing markets, this study underscores the urgency of policy reforms to safeguard the financial well-being of the widowed. For example, policy debates and interventions should focus on maintaining secure housing post-widowhood in such markets. Additionally, establishing financial support systems could mitigate the economic vulnerabilities faced by widows and widowers. These measures are crucial for ensuring that widowed individuals are not overly disadvantaged in today’s economic environment, thereby bolstering their financial security and overall well-being.

## Supplementary Material

gbae116_suppl_Supplementary_Material
