# Supplementary material for: Changes in Household Wealth Over the Process of Widowhood Across European Countries
Source: J Gerontol B Psychol Sci Soc Sci. 2024 Jul 16;79(10):gbae116. doi: 10.1093/geronb/gbae116 (PMC11374882; doi:10.1093/geronb/gbae116)
Supplement: gbae116_suppl_Supplementary_Material [file gbae116_suppl_supplementary_material.docx]

***The Journals of Gerontology, Series B: Psychological Sciences and Social Sciences* Supplementary Material: Kapelle & Van Winkle. Changes in Household Wealth over the Process of Widowhood across European Countries.**

**SHARE wealth data**

Wealth measures are assessed within comprehensive assets and housing models in the SHARE. These models have been included across all regular SHARE waves. By definition, the assets and housing models were not included in wave 3, which only collected SHARELIFE data. Respondents who had not completed the SHARELIFE questionnaire in wave 3, completed it in wave 7 and were not asked the regular survey in that wave. While the assets model is completed by the household’s financial respondent, the housing module is completed by the household respondent. Hence, for single-person households, the financial and household respondent is the main respondent. For couple households, the financial respondent and the household respondent may differ and are one of the partners.

Overall, the assets and housing modules include a vast range of questions regarding various asset and debt components. Nine asset and liability components are covered within the questionnaire. Asset components include information on owner-occupied housing, other property, business assets, vehicles, bank accounts, bonds, stocks, and mutual funds, as well as long-term investments. Liabilities include information on mortgages and other outstanding debt.

To assess each component, the SHARE questionnaire generally asks a filter question first to assess whether a certain wealth component is owned at all. If the respondent confirms ownership, additional questions on the total value of the components are asked. For business assets, the questionnaire additionally asks about the share that is owned by the household, ensuring that data users can capture the value in business assets that is owned by the household rather than individuals outside of the household. Across all wealth components, the respondent is asked to provide ownership status and value amount for the couple level for couples.

Because of the survey design, the assessment of wealth components is consistent across all countries, allowing for a straightforward comparison between the included countries. To provide more user-friendly data and handle missing values in any of the wealth components, the SHARE team imputes wealth data and provides those data as part of the gv_imputations dataset.

**Supplementary Tables**

**Supplementary Table 1** Overview of variables used for the imputation process including number and share of missing values.

|  | Wave 1 | | Wave 2 | | Wave 4 | | Wave 5 | | Wave 6 | | Wave 7 | | Wave 8 | |
| --- | --- | --- | --- | --- | --- | --- | --- | --- | --- | --- | --- | --- | --- | --- |
|  | Nr. | % | Nr. | % | Nr. | % | Nr. | % | Nr. | % | Nr. | % | Nr. | % |
| Housing wealth* | 6076 | 52.67 | 8074 | 49.71 | 12705 | 51.79 | 15443 | 49.78 | 14306 | 47.31 | 4391 | 48.96 | 7624 | 46.63 |
| Non-housing wealth* | 6803 | 58.97 | 8940 | 55.04 | 14240 | 58.04 | 17135 | 55.23 | 15744 | 52.06 | 4823 | 53.77 | 8620 | 52.72 |
| Age at widowhood | None | | | | | | | | | | | | | |
| Marital status* | 0 | 0.00 | 2 | 0.01 | 10 | 0.04 | 9 | 0.03 | 7 | 0.02 | 0 | 0.00 | 0 | 0.00 |
| Gender | None | | | | | | | | | | | | | |
| Age | None | | | | | | | | | | | | | |
| Partner's age* | 2 | 0.02 | 0 | 0.00 | 1 | 0.00 | 1 | 0.00 | 3 | 0.01 | 0 | 0.00 | 0 | 0.00 |
| Years in education* | 90 | 0.78 | 201 | 1.24 | 1204 | 4.91 | 1217 | 3.92 | 1107 | 3.66 | 375 | 4.18 | 518 | 3.17 |
| Partner's years in education* | 1941 | 16.83 | 315 | 1.94 | 1526 | 6.22 | 1080 | 3.48 | 1577 | 5.21 | 360 | 4.01 | 979 | 5.99 |
| Nr of children* | 30 | 0.26 | 53 | 0.33 | 76 | 0.31 | 119 | 0.38 | 147 | 0.49 | 7 | 0.08 | 101 | 0.62 |
| Nr of grandchildren* | 53 | 0.46 | 78 | 0.48 | 115 | 0.47 | 185 | 0.60 | 257 | 0.85 | 32 | 0.36 | 109 | 0.67 |
| Homeowner* | 66 | 0.57 | 81 | 0.5 | 138 | 0.56 | 202 | 0.65 | 342 | 1.13 | 5 | 0.06 | 185 | 1.13 |
| No of rooms | 129 | 1.12 | 180 | 1.11 | 346 | 1.41 | 336 | 1.08 | 302 | 1.00 | 41 | 0.46 | 90 | 0.55 |
| Household income* | 7486 | 64.89 | 9437 | 58.1 | 13369 | 54.49 | 16714 | 53.87 | 14671 | 48.52 | 4180 | 46.6 | 7968 | 48.73 |
| Country | 0 | 0.00 | 0 | 0.00 | 0 | 0.00 | 0 | 0.00 | 0 | 0.00 | 0 | 0.00 | 0 | 0.00 |

*Notes:* Data are from the SHARE survey (release 8.0.0). Asterix indicates variables imputed by the SHARE team.

**Supplementary** **Table 2** Cell sizes across the widowhood process across countries.

| Widowhood process categories | Country | | | | | | | | | | | |
| --- | --- | --- | --- | --- | --- | --- | --- | --- | --- | --- | --- | --- |
|  | Austria | Germany | Sweden | Spain | Italy | France | Denmark | Switzerland | Belgium | Czech Republic | Poland | Total |
| Continuously married | 9039 | 12271 | 11907 | 15004 | 14399 | 12405 | 10123 | 8503 | 14983 | 11524 | 4336 | 124494 |
| Married, >3 | 273 | 399 | 476 | 591 | 546 | 464 | 358 | 267 | 454 | 383 | 226 | 4437 |
| -2/3 | 113 | 135 | 159 | 258 | 205 | 162 | 154 | 117 | 186 | 199 | 123 | 1811 |
| -1 | 90 | 94 | 88 | 187 | 122 | 110 | 92 | 77 | 119 | 151 | 62 | 1192 |
| 0 | 70 | 115 | 92 | 159 | 118 | 123 | 84 | 78 | 93 | 132 | 67 | 1131 |
| 1 | 109 | 120 | 132 | 227 | 151 | 120 | 124 | 92 | 152 | 182 | 77 | 1486 |
| 2/3 | 106 | 121 | 154 | 230 | 190 | 154 | 138 | 110 | 162 | 185 | 142 | 1692 |
| 4/6 | 69 | 123 | 159 | 192 | 166 | 139 | 157 | 91 | 154 | 137 | 149 | 1536 |
| >6 | 71 | 91 | 152 | 148 | 105 | 124 | 95 | 70 | 119 | 73 | 69 | 1117 |
| Total | 9940 | 13469 | 13319 | 16996 | 16002 | 13801 | 11325 | 9405 | 16422 | 12966 | 5251 | 138896 |

*Notes:* Data are from the SHARE survey (release 8.0.0)

**Supplementary Table 3** Cell sizes across the widowhood process across countries (women only).

| Widowhood process categories | Country | | | | | | | | | | | |
| --- | --- | --- | --- | --- | --- | --- | --- | --- | --- | --- | --- | --- |
|  | Austria | Germany | Sweden | Spain | Italy | France | Denmark | Switzerland | Belgium | Czech Republic | Poland | Total |
| Continuously married | 4312 | 6020 | 5840 | 7419 | 7051 | 6008 | 4982 | 4127 | 7316 | 5626 | 2174 | 60875 |
| Married, >3 | 207 | 267 | 324 | 444 | 421 | 346 | 232 | 184 | 284 | 277 | 157 | 3143 |
| -2/3 | 81 | 84 | 110 | 192 | 155 | 121 | 99 | 79 | 119 | 145 | 90 | 1275 |
| -1 | 61 | 59 | 65 | 143 | 95 | 75 | 65 | 49 | 83 | 124 | 45 | 864 |
| 0 | 56 | 80 | 59 | 124 | 91 | 87 | 56 | 58 | 61 | 94 | 52 | 818 |
| 1 | 77 | 79 | 95 | 178 | 115 | 89 | 82 | 60 | 109 | 144 | 47 | 1075 |
| 2/3 | 83 | 78 | 112 | 180 | 152 | 106 | 92 | 69 | 110 | 144 | 100 | 1226 |
| 4/6 | 52 | 76 | 110 | 164 | 131 | 96 | 111 | 61 | 106 | 115 | 107 | 1129 |
| >6 | 50 | 57 | 120 | 124 | 78 | 95 | 61 | 44 | 99 | 65 | 58 | 851 |
| Total | 4979 | 6800 | 6835 | 8968 | 8289 | 7023 | 5780 | 4731 | 8287 | 6734 | 2830 | 71256 |

*Notes:* Data are from the SHARE survey (release 8.0.0)

**Supplementary Figures**

**Supplementary Figure 1** Fixed-effects regression coefficients for household net wealth (ihs-transformed) through widowhood across 11 countries, excluding wave 8


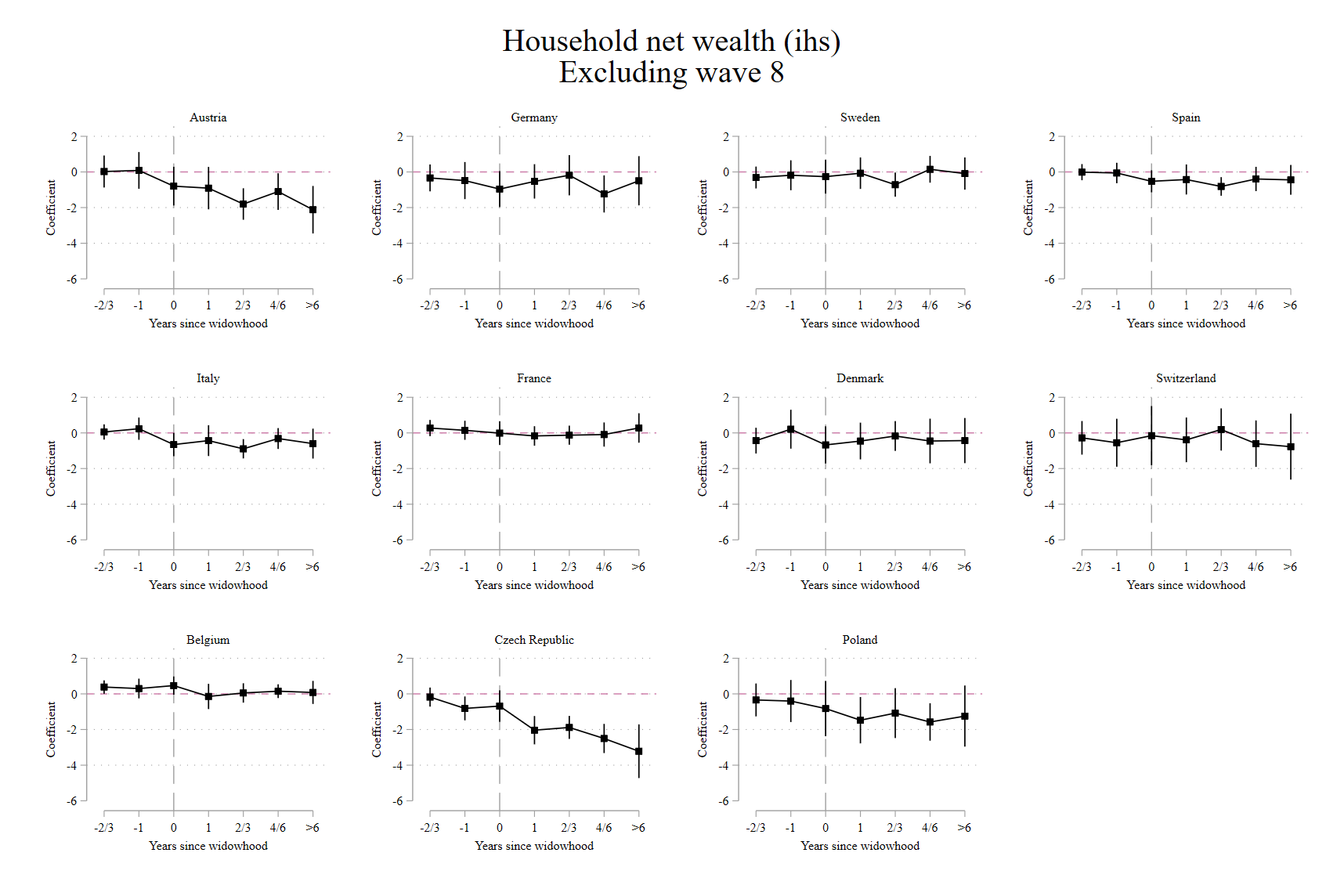
*Notes:* Reference “married and at least four years prior to widowhood”. Whiskers indicate 95% confidence intervals. Data are from the SHARE release 8.0.0 (waves 1 to 8; unweighted; multiply imputed).

**Supplementary Figure 2** Fixed-effects regression coefficients for household net housing and non-housing wealth across 11 countries, excluding wave 8


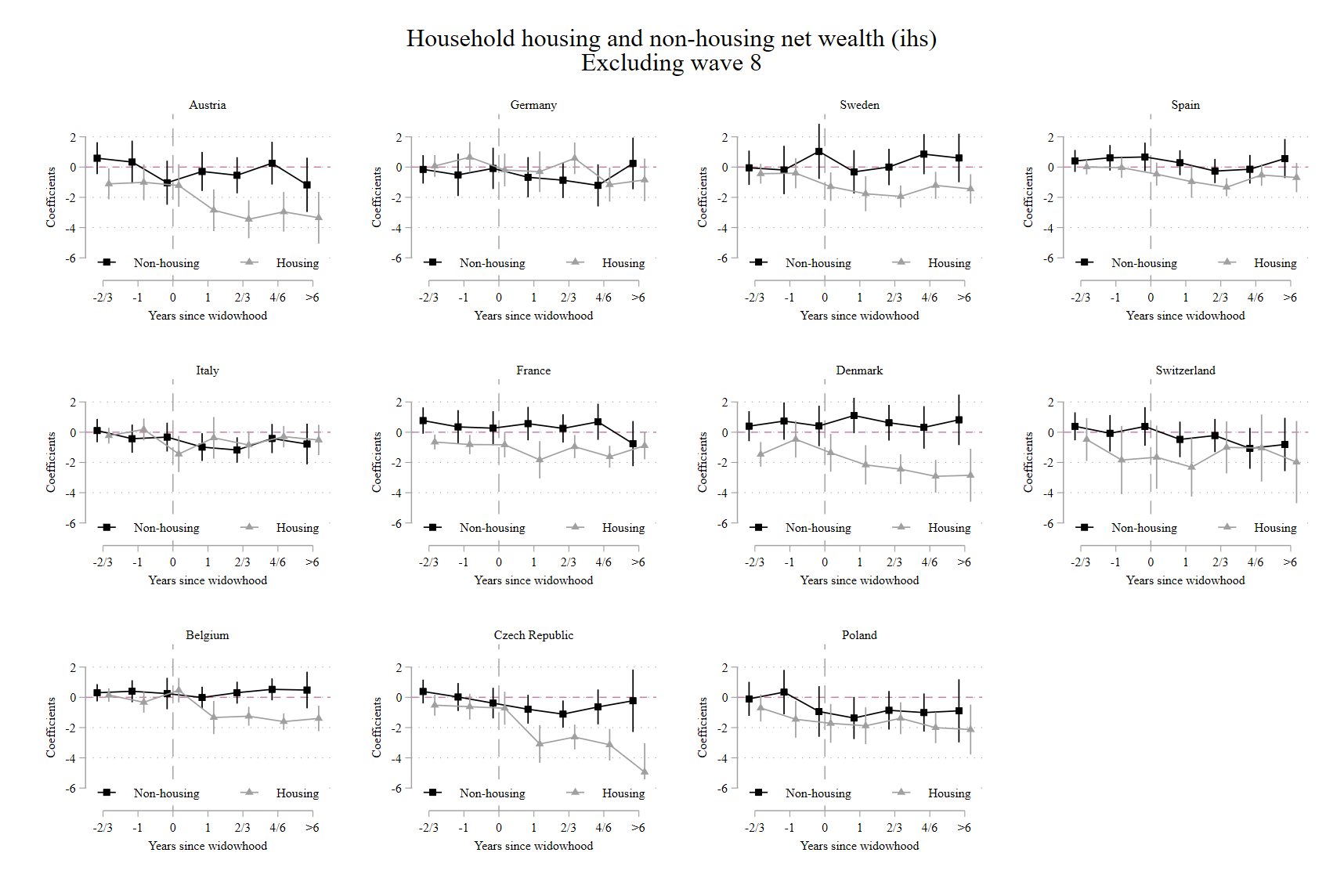


*Notes:* Reference “married and at least four years prior to widowhood”. Whiskers indicate 95% confidence intervals. Data are from the SHARE release 8.0.0 (waves 1 to 8; unweighted; multiply imputed).

**Supplementary Figure 3** Fixed-effects regression coefficients for household net wealth (ihs-transformed) through widowhood across 11 countries, including potential mechanisms as covariates


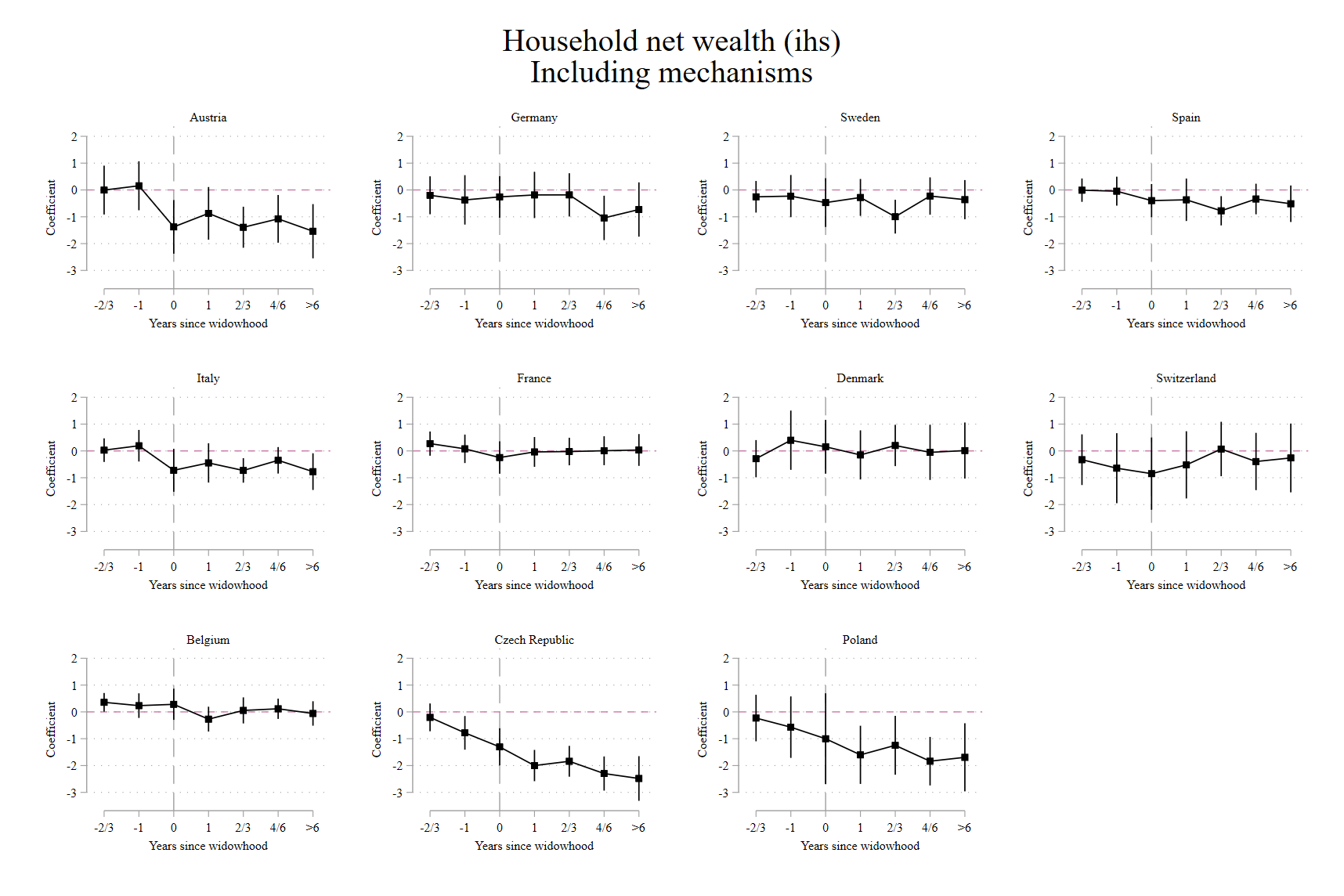
*Notes:* Reference “married and at least four years prior to widowhood”. Whiskers indicate 95% confidence intervals. Data are from the SHARE release 8.0.0 (waves 1 to 8; unweighted; multiply imputed).

**Supplementary Figure 4** Fixed-effects regression coefficients for household net housing and non-housing wealth across 11 countries, including potential mechanisms as covariates


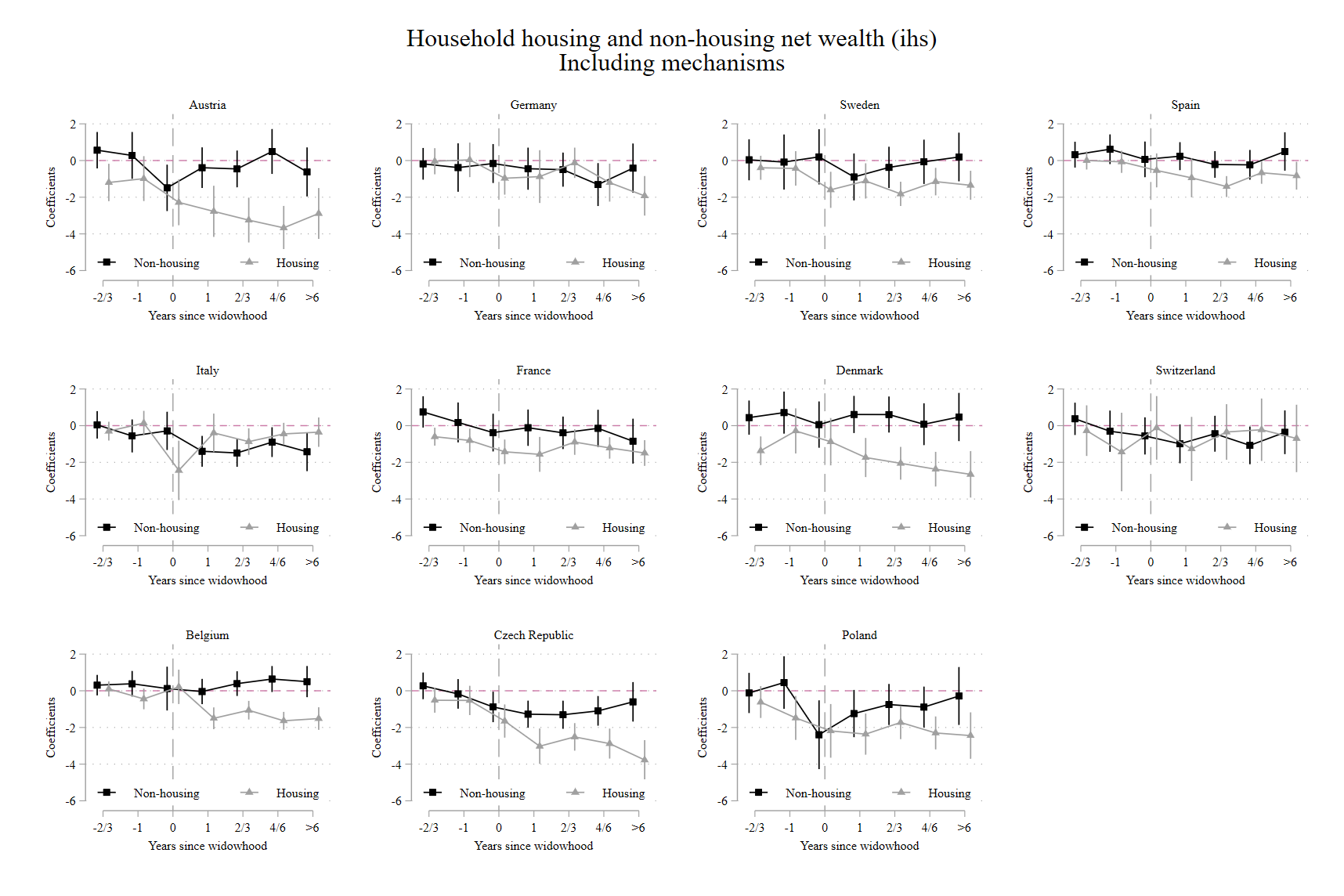
*Notes:* Reference “married and at least four years prior to widowhood”. Whiskers indicate 95% confidence intervals. Data are from the SHARE release 8.0.0 (waves 1 to 8; unweighted; multiply imputed).

**Supplementary Figure 5** Fixed-effects regressions of the number of rooms in the dwelling across 11 countries


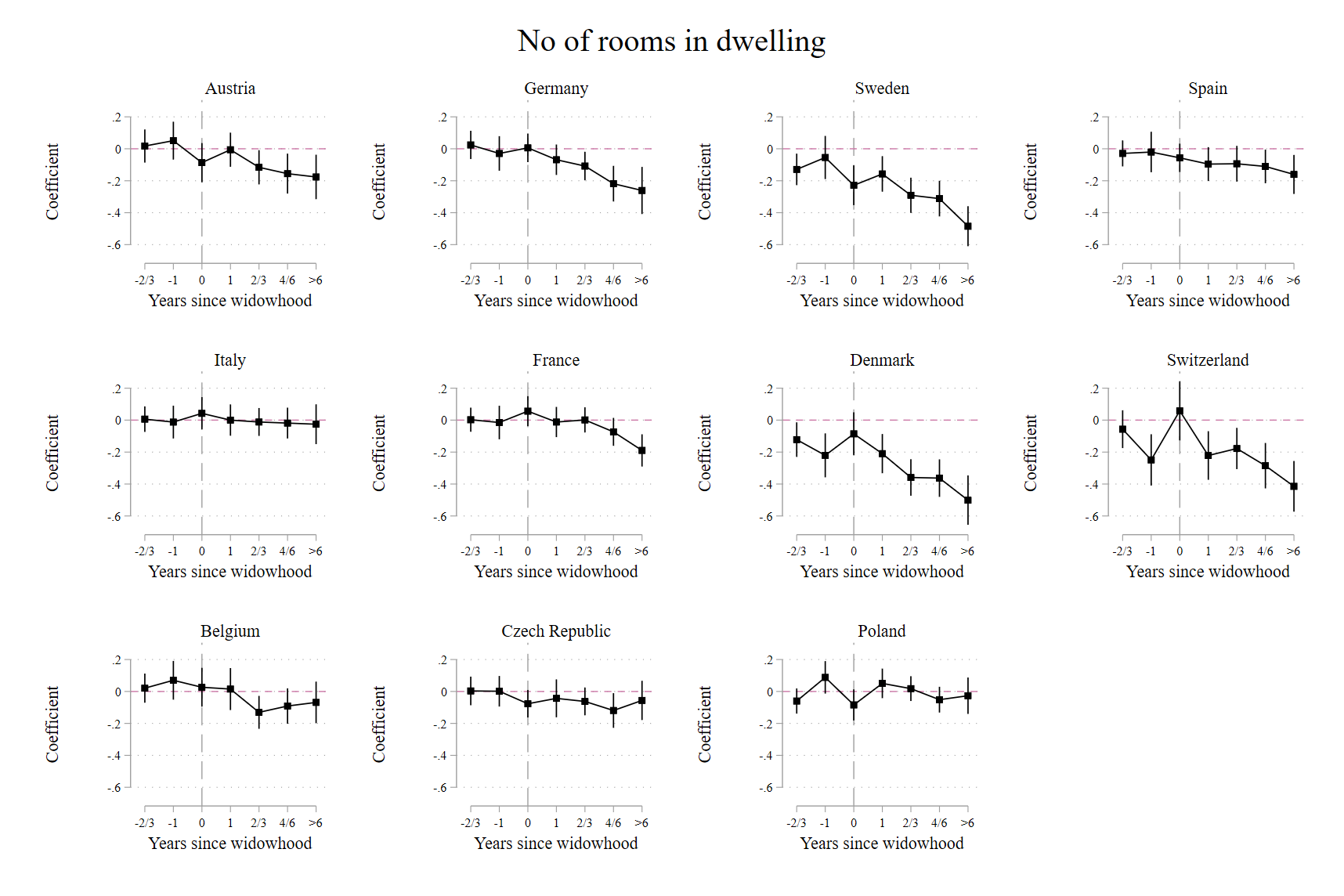
*Notes:* Reference “married and at least four years prior to widowhood”. Whiskers indicate 95% confidence intervals. Data are from the SHARE release 8.0.0 (waves 1 to 8; unweighted; multiply imputed).

**Supplementary Figure 6** Fixed-effects regressions of the likelihood of homeownership across 11 countries


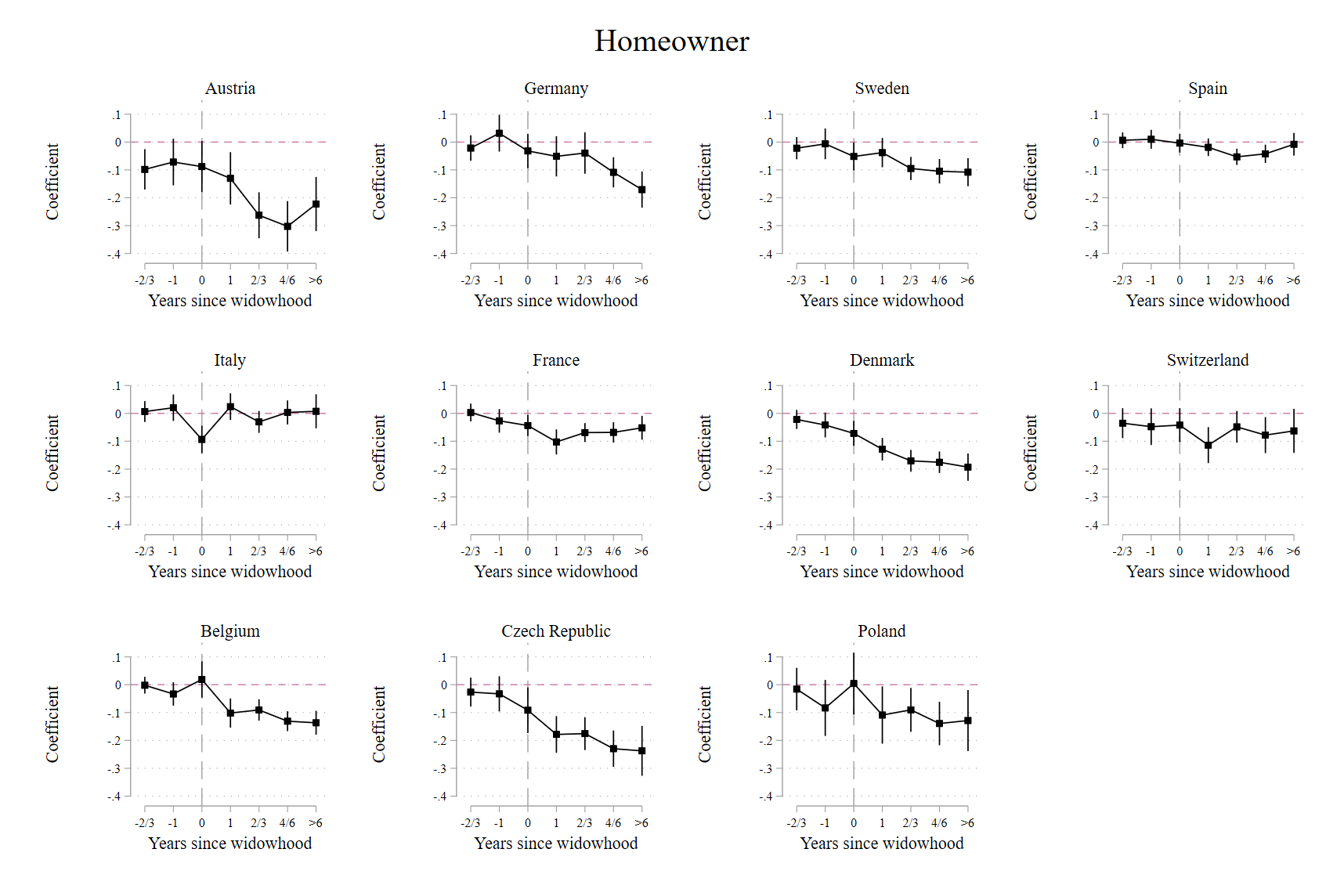
*Notes:* Reference “married and at least four years prior to widowhood”. Whiskers indicate 95% confidence intervals. Data are from the SHARE release 8.0.0 (waves 1 to 8; unweighted; multiply imputed).

**Supplementary Figure 7** Fixed-effects regression coefficients for household net wealth (ihs-transformed) through widowhood across 11 countries, women only


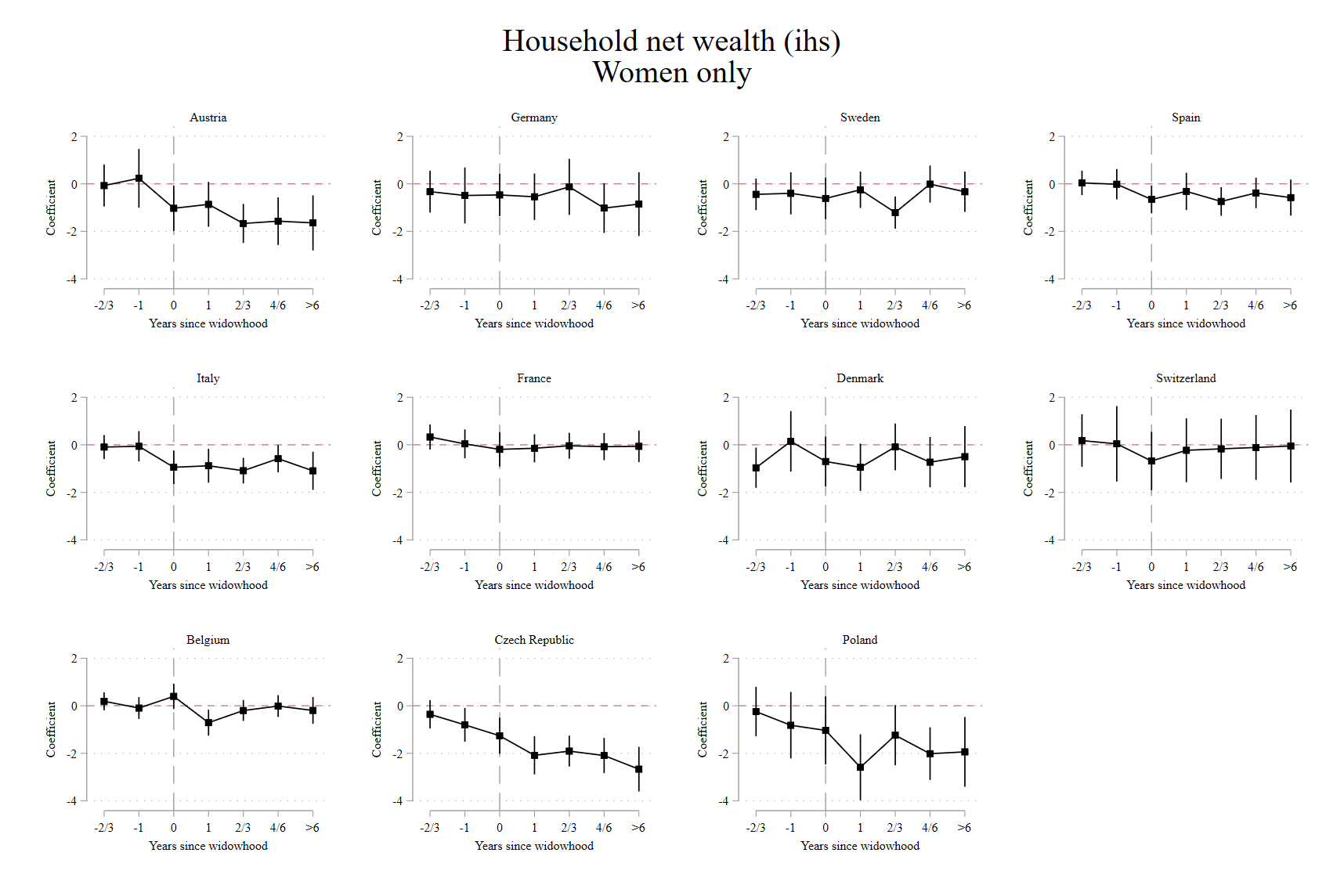
*Notes:* Reference “married and at least four years prior to widowhood”. Whiskers indicate 95% confidence intervals. Data are from the SHARE release 8.0.0 (waves 1 to 8; unweighted; multiply imputed).

**Supplementary Figure 8** Fixed-effects regression coefficients for household net housing and non-housing wealth across 11 countries, women only


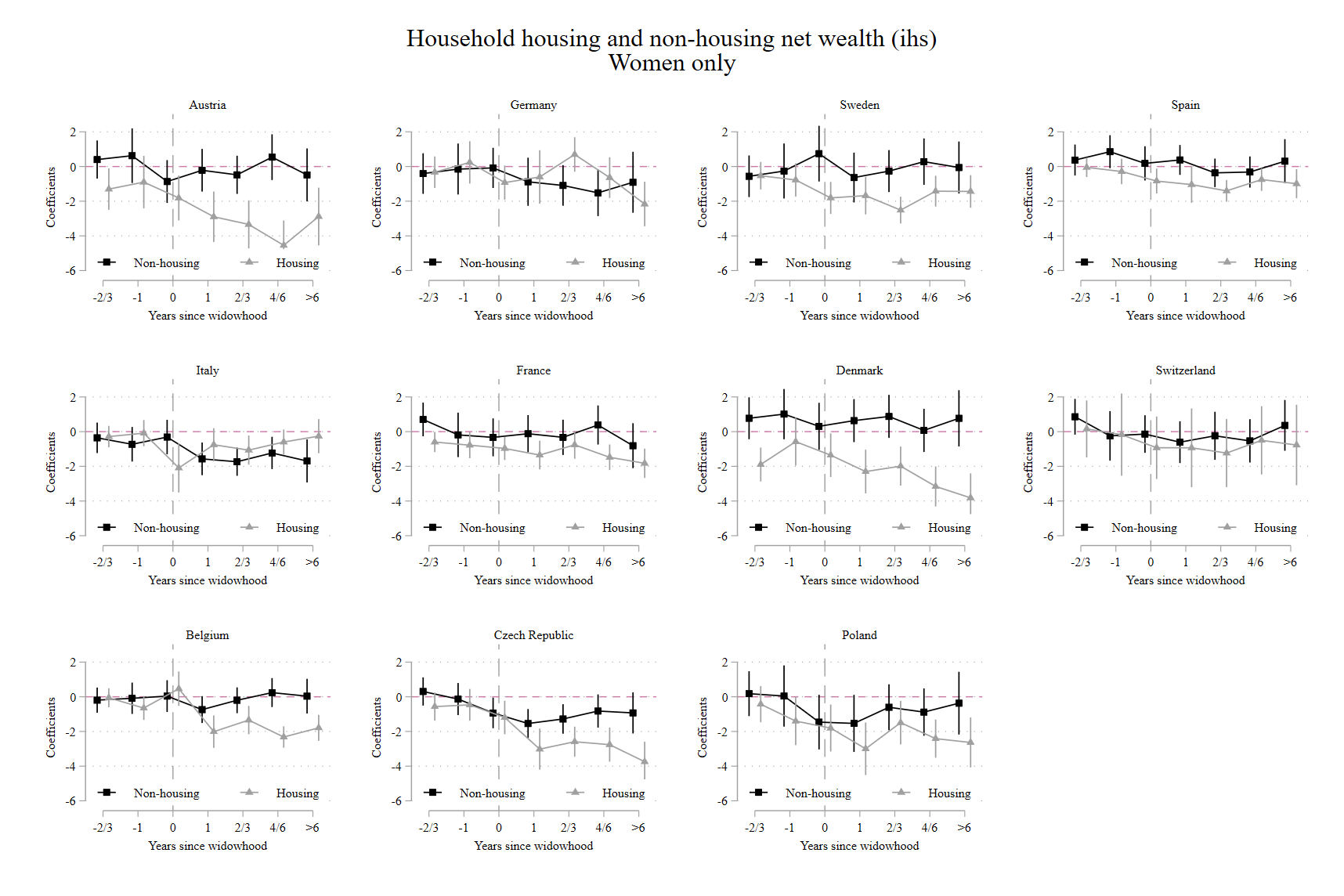
*Notes:* Reference “married and at least four years prior to widowhood”. Whiskers indicate 95% confidence intervals. Data are from the SHARE release 8.0.0 (waves 1 to 8; unweighted; multiply imputed).

**Supplementary Figure 9** Fixed-effects regression coefficients for net wealth (ihs-transformed) through widowhood across 11 countries, per capita wealth


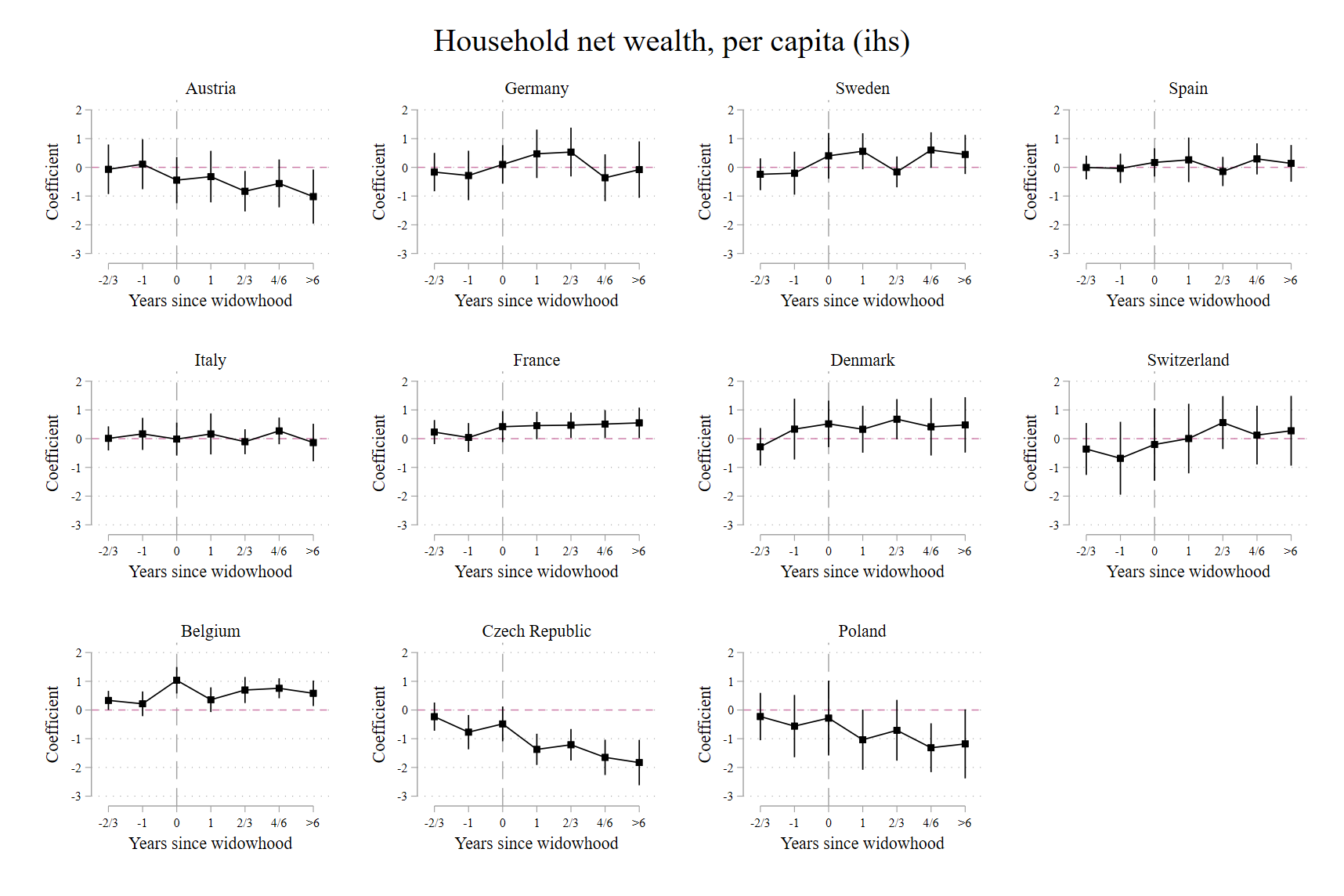
*Notes:* Reference “married and at least four years prior to widowhood”. Whiskers indicate 95% confidence intervals. Data are from the SHARE release 8.0.0 (waves 1 to 8; unweighted; multiply imputed).

**Supplementary Figure 10** Fixed-effects regression coefficients for net housing and non-housing wealth across 11 countries, per capita wealth


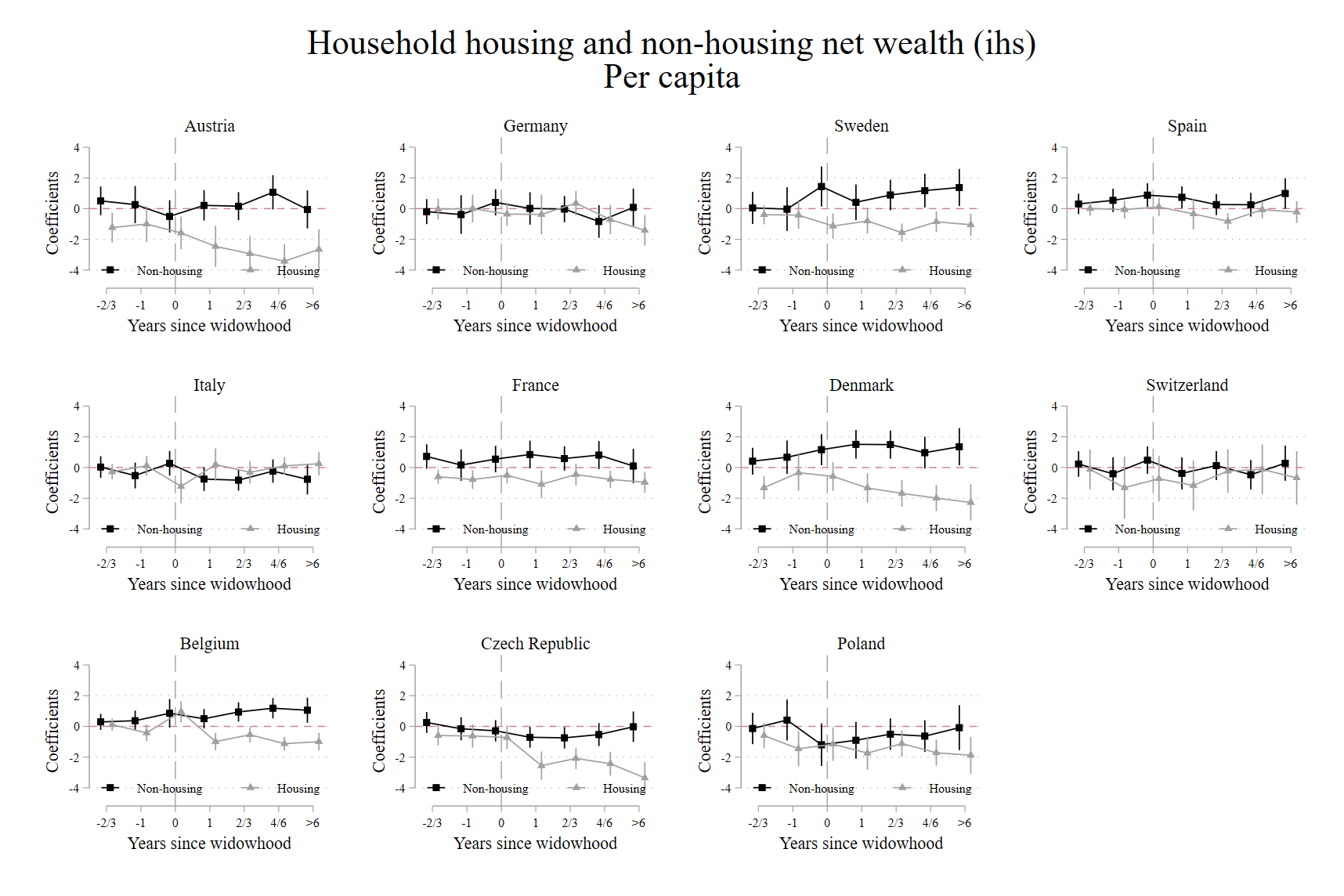
*Notes:* Reference “married and at least four years prior to widowhood”. Whiskers indicate 95% confidence intervals. Data are from the SHARE release 8.0.0 (waves 1 to 8; unweighted; multiply imputed).

**Supplementary Figure 11** Fixed-effects regression coefficients for household net wealth (ihs-transformed) through widowhood across 11 countries, using non-imputed data


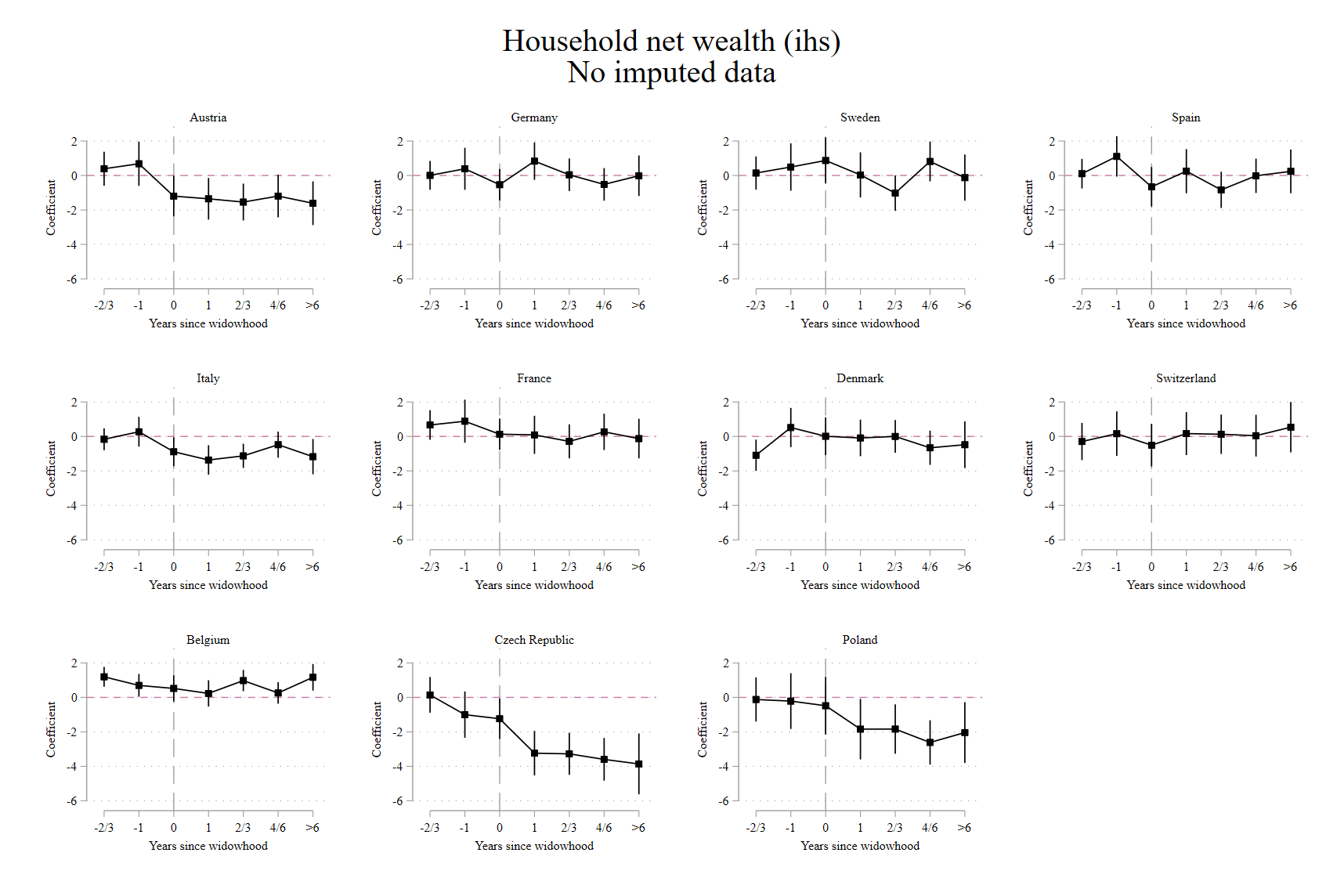
*Notes:* Reference “married and at least four years prior to widowhood”. Whiskers indicate 95% confidence intervals. Data are from the SHARE release 8.0.0 (waves 1 to 8; unweighted; multiply imputed).

**Supplementary Figure 12** Fixed-effects regression coefficients for household net housing and non-housing wealth across 11 countries, using non-imputed data


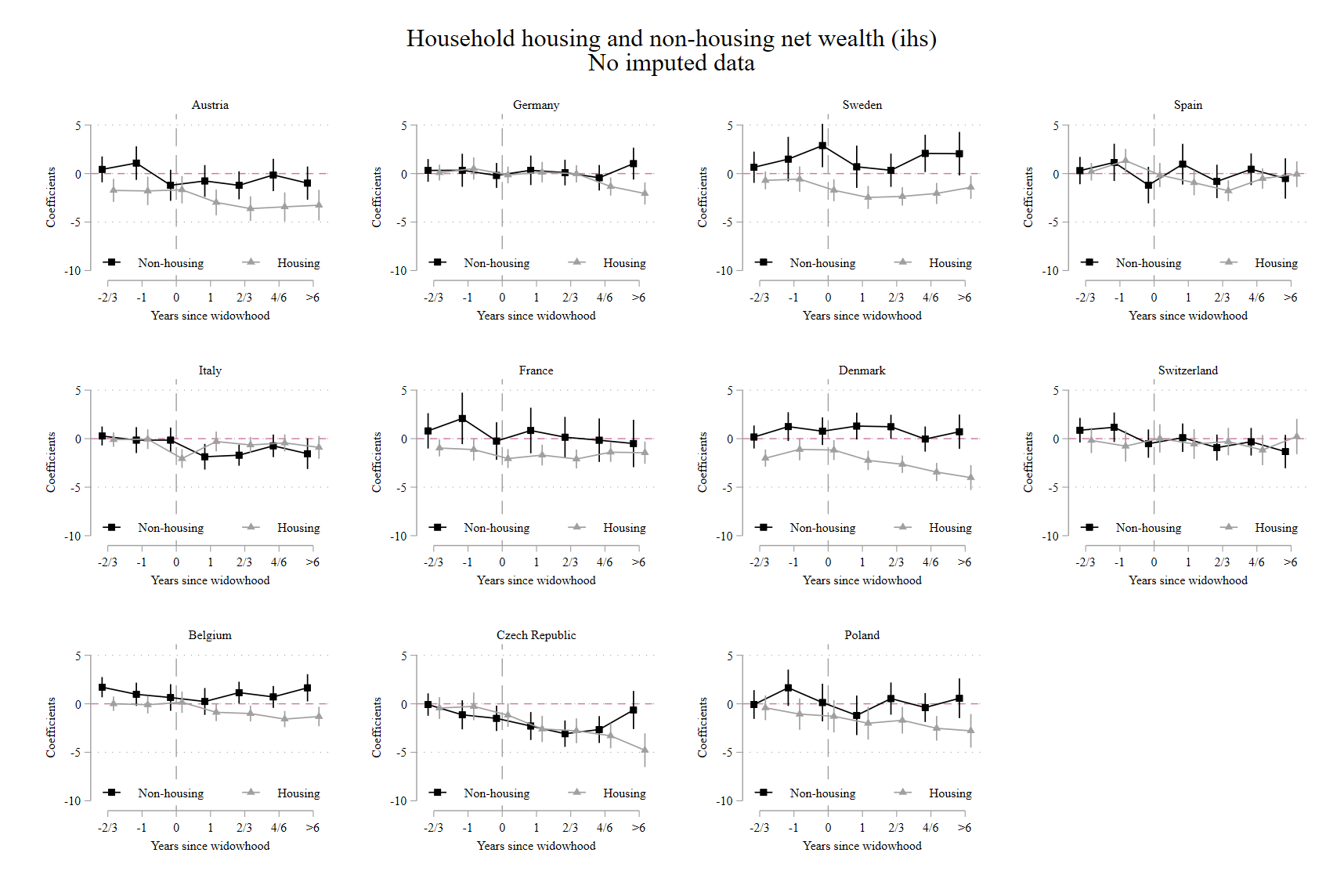
*Notes:* Reference “married and at least four years prior to widowhood”. Whiskers indicate 95% confidence intervals. Data are from the SHARE release 8.0.0 (waves 1 to 8; unweighted; multiply imputed).
